# Maternal and foetal complications of pregestational and gestational diabetes: a descriptive, retrospective cohort study

**DOI:** 10.1038/s41598-024-59465-x

**Published:** 2024-04-19

**Authors:** Miriam Oros Ruiz, Daniel Perejón López, Catalina Serna Arnaiz, Júlia Siscart Viladegut, Joan Àngel Baldó, Joaquim Sol

**Affiliations:** Institut Català de La Salut, Lleida, Spain

**Keywords:** Pregnancy, Gestational diabetes, Prevalence, Prematurity, Caesarean, Apgar, Macrosomia, Adverse neonatal outcomes, Pregestational diabetes, Public health, Endocrinology, Health care

## Abstract

Gestational diabetes is characterized by hyperglycaemia diagnosed during pregnancy. Gestational and pregestational diabetes can have deleterious effects during pregnancy and perinatally. The baby's weight is frequently above average and might reach macrosomia (≥ 4 kg), which can reduce pregnancy time causing preterm births, and increase foetal-pelvic disproportion which often requires delivery by caesarean section. Foetal-pelvic disproportion due to the baby’s weight can also cause foetal distress resulting in lower Apgar scores. To analyse the association between pregestational and gestational diabetes with maternal and foetal risk. We conducted a retrospective cohort study in women pregnant between 2012 and 2018 in the region of Lleida. Regression coefficients and 95% confidence intervals (CI) were used. The multivariate analysis showed statistically significant associations between pregestational diabetes and: prematurity (OR 2.4); caesarean section (OR 1.4); moderate (OR 1.3), high (OR 3.3) and very high (OR 1.7) risk pregnancies; and birth weight ≥ 4000 g (macrosomia) (OR 1.7). In getational diabetes the multivariate analysis show significant association with: caesarean section (OR 1.5); moderate (OR 1.7), high (OR 1.7) and very high (OR 1.8) risk pregnancies and lower 1-minuto Apgar score (OR 1.5). Pregestational and gestational diabetes increase: pregnancy risk, caesarean sections, prematurity, low Apgar scores, and macrosomia.

## Introduction

Gestational diabetes (GD) is defined by hyperglycaemia diagnosed during pregnancy^1^. Maternal factors associated with gestational diabetes are increasing, mainly: the rise of overweight and obesity in women, risks factors to develop type 2 diabetes and gestational diabetes; and the older average age of the mothers in the pregnancy of their first child.

GD is the most common pregnancy-associated disorder, with potential obstetric and perinatal consequences. Therefore, antenatal care in women with GD require hospital specialists instead of primary care health checks.

A study published in 2021 by Wdowiak et al.^2^ showed that overweight of the mother before pregnancy correlates with high birth weight of the baby. This was confirmed in the review by Catalano et al.^3^. A study on hyperglycaemia and adverse pregnancy outcomes by Metzger et al.,^4^ which comprised data from over 23,000 women, found that the prevalence of macrosomia was 6.7%, 10.2% and 20.2% in 17,244 non-obese women without GD, 2,791 non-obese women with GD, and 935 obese women with GD, respectively. Women with obesity without GD had a 13.6% higher risk of macrosomia (defined as a birth weight of 4000 g and over) than non-obese women. Adverse pregnancy outcomes are more common in women with pregestational diabetes compared to GD, according to a systematic review by Malaza et al. Complications include cesarean section, preterm birth, congenital anomalies, pre-eclampsia, neonatal hypoglycemia, macrosomia, neonatal intensive care unit admission, stillbirth, Apgar score, large for gestational age, induction of labor, respiratory distress syndrome, and miscarriages^5^.

Insulin is an anabolic hormone that regulates foetal growth^6,7^. Maternal hyperglycaemia induces hyperglycaemia and hyperinsulinemia in the foetus. This stimulates anabolism and consequently the development of muscle, adipose, and connective tissue. The combination of hyperglycaemia and hyperinsulinemia cause an increase in storage of foetal fat and protein which results in macrosomia^6^.

Macrosomia is defined by a foetal weight by gestational age above the 90th percentile, or equal/over 4000 g^8^. This weight can shorten pregnancy time causing prematurity. It can also cause foetal-pelvic disproportion, which requires more caesarean sections and can result in lower Apgar scores.

The common etiological mechanism in pregestational and gestational diabetes is insulin resistance. Both cause hyperglycaemia in pregnancy and both have been associated with adverse effects in pregnancy. With this study conducted in the health region of Lleida, we aim to analyse the prevalence of pregestational and gestational diabetes, of high-risk pregnancies and of complications in the baby.

## Methodology

### Design and data collection

Retrospective, cohort observational study in pregnant women between 2012 and 2018 in the health region of Lleida.

Data from women who had delivered at the hospital Arnau de Vilanova between 1-1-2012 and 31-12-2018 were obtained through the CMBD database (Minimum Data Set) of the electronic medical records database e-CAP, and from electronic prescriptions of the Catalan Health Service.

This study is part of the ILERPREGNANT project. The main objective of ILERPREGNANT is to analyse the prevalence of different conditions, therapeutic prescription and pharmacological adherence during pregnancy^9^.

### Participants

Women who had delivered between 1-1-2012 and 31-12-2018. Pregnancy data are included from the date of the last period until the date of birth. As such, data from 2011 were taken into account for pregnant women with a birth date in 2012 but with a last period date in 2011. Pregnant women who do not belong to the health region of Lleida were excluded. To verify the representativity of the sample, the percentage of births studied (births registered at the University Hospital Arnau de Vilanova in Lleida) was calculated with respect to the total number of births in the health region of Lleida according to the data obtained from the Statistical Institute of Catalonia (Idescat) database (Table 1).

**Table 1:** Number of deliveries registered in the health region of Lleida by years and number of deliveries of the sample studied with the percentage they represent.

Year	Idescat deliveries	Sample deliveries	Idescat/sample (%)
2012	3788	3635	90
2013	3535	3370	89
2014	3592	3308	86
2015	3426	3162	86
2016	3283	3180	90
2017	3197	3034	88
2018	3029	3001	93

The main variable recorded was presence of gestational diabetes or previous diabetes, a dichotomous qualitative variable defined by an abnormal O'Sullivan test at weeks 24 and 28 of pregnancy (according to GEDE, 2014), verified by an Oral Glucose Tolerance test (OGT). Extraction of medical records in e-CAP with the code for gestational diabetes (ICD-10 code O24.9).

Other variables taken into account were: risk of the pregnancy; duration of the pregnancy (miscarriage, preterm, term, prolonged); caesarean section; birth weight (< 2500 g = underweight, between 2500 g and 3999 g = normal weight, and ≥ 4000 g = macrosomia), 1-minute and 5-minute Apgar score; and preeclampsia^9^.

### Ethics

This study was approved by the Clinical Research Ethics Committee (CREC) of the *Institut de Recerca IDIAP Jordi Gol* (code 19/194-P). It follows the tenets of the Declaration of Helsinki. The information was extracted from centralized medical files in the e-CAP database by the Health Research and Assessment Management Department. Informed consent from participants was not needed. The variables in the e-CAP database were processed anonymously and with all the guarantees of confidentiality established by the National Law and Regulation 2016/679 of the European Parliament and Council on the protection of individuals with regard to the use of personal information. The need of Informed consent was waived by “Idiap Jordi Gol i Gurina comitè”.

### Human and animal rights

All procedures followed were in accordance with the ethical standards of the responsible committee on human experimentation (institutional and national) and with the Helsinki Declaration of 1975, as revised in 2008.

### Informed consent

The databases from which the data were obtained are based on opt-out presumed consent and data are anonymized. If a patient decides to opt out, their data is excluded from the database. The need of Informed consent was waived by the Clinical Research Ethics Committee (CREC) of the Institut de Recerca IDIAP Jordi Gol.

## Results

### Study population

**Figure 1:**
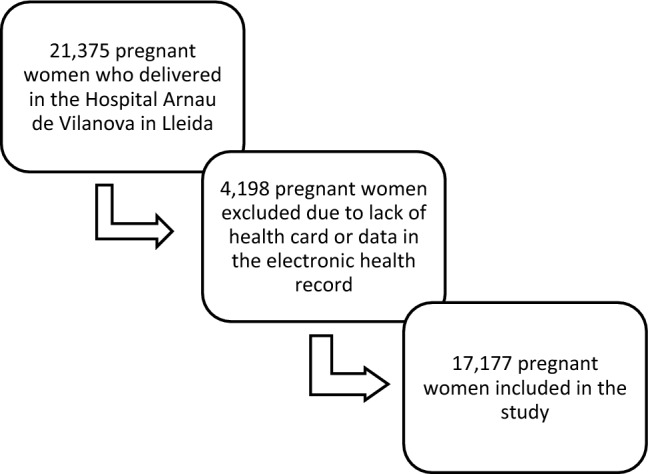
Selection process of participating pregnant women.

The sample consisted of 21,375 pregnant women who had given birth at the Hospital Arnau de Vilanova in Lleida between 2012 and 2018 (both included). Women who did not have a personal identification code (CIP) (n = 1625) were excluded from the group, as well as women with insufficient data in the clinical record. The final sample consisted of 17,177 patients (Fig. 1).

### Characteristics of the study population

The prevalence of pregnant women with diabetes was 8.2%, 1.6% pregestational (diabetes mellitus type 1 N = 4, diabetes mellitus type 2 N = 271) and 6.6% gestational (N = 1123). A total of 87.2% pregnancies ended in term deliveries, 5.9% were preterm infants, 2.5%, prolonged pregnancies, and 4.4% ended in miscarriage. Preeclampsia occurred in 0.9% of pregnancies. Caesarean section was performed in 17.3% deliveries. Most babies (87.2%) had a normal weight at birth (2500–4000 g), 6% had low weight (< 2500 g), and 6.8% had macrosomia (> 4000 g). Low 1-min and 5-min Apgar scores (< 7) were found in 2.5% and 0.8% infants, respectively (Table 2).

**Table 2:** Characteristics of the study population.

	N = 17,177
Year of pregnancy	17,177
2011	43 (0.2%)
2012	2740 (16%)
2013	2525 (14.7%)
2014	2491 (14.5%)
2015	2419 (14.1%)
2016	2418 (14.1%)
2017	2317 (13.5%)
2018	2224 (12.9%)
Age at pregnancy	17,177
< 30	6981 (40.6%)
30–35	4463 (26%)
> 35	5733 (33.4%)
BMI	16,803
≤ 25	11,117 (66.2%)
26–30	3700 (22%)
≥ 30	1986 (11.8%)
Number of pregnancy	17,177
1	9009 (52.4%)
2	5181 (30.2%)
3	1870 (10.9%)
4	646 (3.8%)
> 4	471 (2.7%)
Preeclampsia	17,177
No	17,018 (99.1%)
Yes	159 (0.9%)
Multiple pregnancy	17,177
No	17,145 (99.8%)
Yes	32 (0.2%)
Caesarean section	17,177
No	14,201 (82.7%)
Yes	2976 (17.3%)
Duration of pregnancy (qualitative)	12,962
Miscarriage	569 (4.4%)
Preterm	769 (5.9%)
Term	11,296 (87.2%)
Post-term	328 (2.5%)
At risk pregnancy	15,333
No risk	7578 (49.4%)
Moderate risk	4527 (29.5%)
High risk	2912 (19%)
Very high risk	316 (2.1%)
Diabetes mellitus (pre-gestational + gestational)	17,177
No	15,773 (91.8%)
Yes	1404 (8.2%)
Gestational diabetes	17,177
No	16,044 (93.4%)
Yes	1133 (6.6%)
Hypothyroidism	17,177
No	16,050 (93.4%)
Yes	1127 (6.6%)
Hypertension	17,177
No	16,778 (97.7%)
Yes	399 (2.3%)
Dyslipidaemia	17,177
No	16,990 (98.9%)
Yes	187 (1.1%)
Depression	17,177
No	16,741 (97.5%)
Yes	436 (2.5%)
Region	15,006
Africa	840 (5.6%)
Latin America	717 (4.8%)
Asia and Middle East	222 (1.5%)
Europe	9461 (63.0%)
Eastern Europe	1533 (10.2%)
Maghreb	2233 (14.9%)
Birth weight	15,133
Low birth weight	910 (6%)
Normal birth weight	13,203 (87.2%)
Macrosomia	1020 (6.8%)
1-min Apgar	15,085
Apgar ≥ 7	14,970 (97.5%)
Apgar < 7	379 (2.5%)
5-min Apgar	15,087
Apgar ≥ 7	14,970 (99.2%)
Apgar < 7	117 (0.8%)

### Factors associated with diabetes during pregnancy

The following factors were associated with diabetes during pregnancy:

The proportion of caesarean sections was higher in women with gestational diabetes 25.6%) or pregestational diabetes (24%) than in women with no diabetes (16.6%). Preterm pregnancies were 7.7% in women with gestational diabetes, 12.8% in pregestational diabetes compared to 5.7% in women with no diabetes. A larger proportion of babies with a birth weight over 4000 g (macrosomia) had mothers with diabetes (8.4% gestational diabetes and 11.8% pregetational diabetes), compared to mothers with no diabetes (6.5%). The prevalence of 1-min Apgar < 7 was of 3.9% in case of mothers with gestational diabetes, 4.1% in case of pregestational diabetes and 2.4% in case of mothers without diabetes (Table 3).

**Table 3:** Risk of maternal and perinatal outcomes by maternal diabetes status.

	No diabetes	Pregestational diabetes	Gestational diabetes	p
	N = 15,773	N = 271	N = 1123	
Age at pregnancy				< 0.001
< 30	6675 (42.3%)	42 (15.5%)	262 (23.3%)	
30–35	5965 (37.8%)	124 (45.8%)	448 (39.9%)	
> 35	3133 (19.9%)	105 (38.7%)	413 (36.8%)	
BMI				< 0.001
≤ 25	9574 (62.1%)	103 (39.3%)	433 (39.2%)	
26–30	3748 (24.3%)	79 (30.2%)	346 (31.3%)	
> 30	2105 (13.6%)	80 (30.5%)	325 (29.4%)	
Number of pregnancy				< 0.001
1	8353 (53.0%)	75 (27.7%)	576 (51.3%)	
2	4743(30.1%)	114 (42.1%)	322 (28.7%)	
3	1682 (10.7%)	48 (17.7%)	138 (12.3%)	
4	573 (3.6%)	20 (7.38%)	52 (4.63%)	
> 4	422 (2.6%)	14 (5.17%)	35 (3.12%)	
Pre-eclampsia				0.467
No	15,630 (99.1%)	1388 (98.9%)	1112 (99.0%)	
Yes	143 (0.9%)	16 (1.1%)	11 (0.98%)	
Caesarean section				< 0.001
No	13,153 (83.4%)	206 (76.0%)	835 (74.4%)	
Yes	2620 (16.6%)	65 (24.0%)	288 (25.6%)	
Duration of pregnancy				< 0.001
Miscarriage	556 (4.7%)	10 (5.10%)	3 (0.34%)	
Preterm	675(5.7%)	25 (12.8%)	69 (7.74%)	
Term	10,327 (87%)	158 (80.6%)	801 (89.9%)	
Post-term	307 (2.6%)	3 (1.53%)	18 (2.02%)	
At risk pregnancy				< 0.001
No risk	7148 (50.8%)	55 (22.6%)	371 (36.9%)	
Moderate risk	4127 (29.3%)	52 (21.4%)	347 (34.5%)	
High risk	2570 (18.3%)	83 (34.2%)	257 (25.6%)	
Very high risk	231 (1.64)	53 (21.8%)	30 (2.99%)	
Weight at birth				0.002
Low birth weight	826 (6%)	16 (7.27%)	68 (6.38%)	
Normal birth weight	12,108 (87.5%)	178 (80.9%)	907 (85.2%)	
Macrosomia	904 (6.5%)	26 (11.8%)	90 (8.45%)	
1-min Apgar				0.001
Apgar ≥ 7	13,465 (97.6%)	209 (95.9%)	1022 (96.1%)	

**Figure 2:**
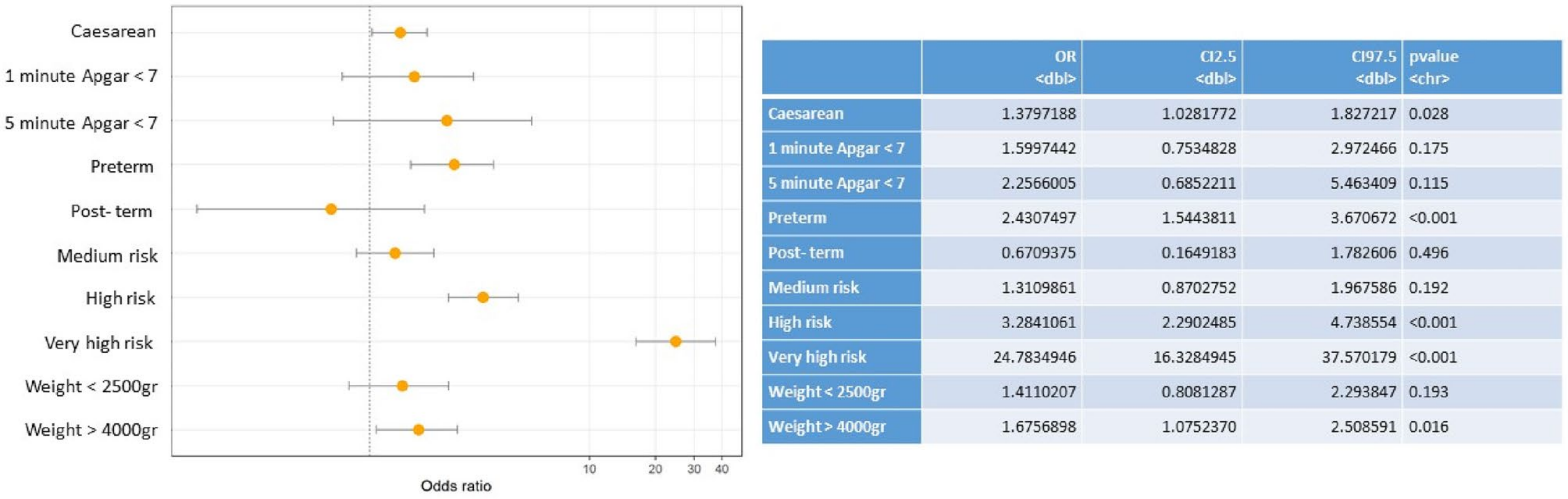
Multivariate analysis of pregestational diabetes and outcomes in the mother and baby.

**Figure 3:**
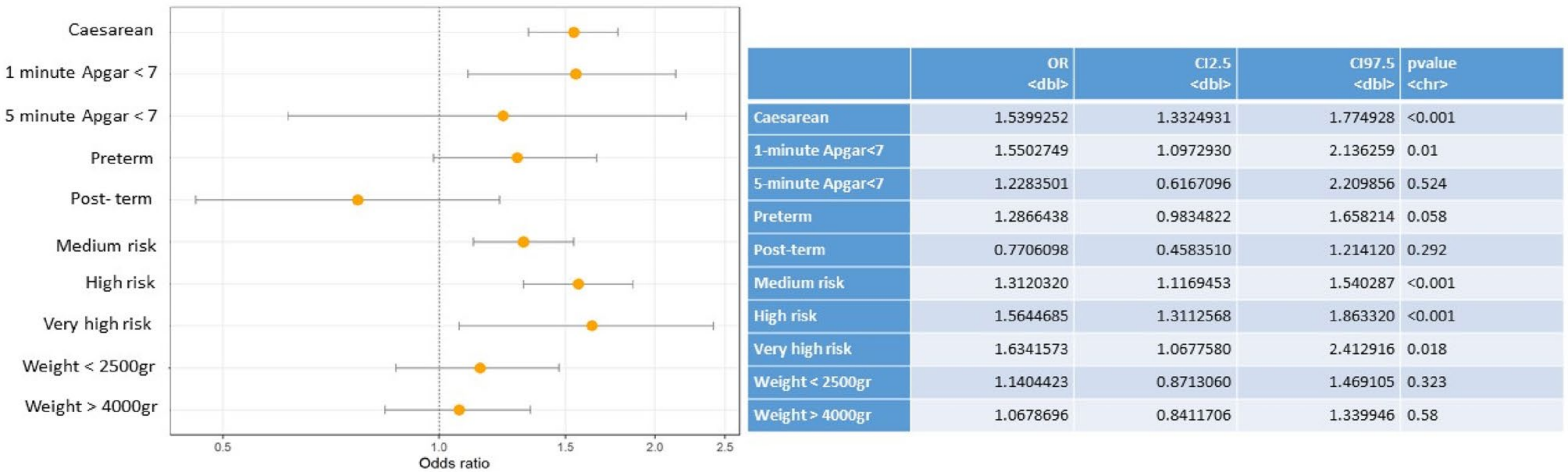
Multivariate analysis of gestational diabetes and outcomes in the mother and baby.

The multivariate analysis showed statistically significant associations between pregestational diabetes and: prematurity (OR 2.4); caesarean section (OR 1.4); moderate (OR 1.3), high (OR 3.3) and very high (OR 1.7) risk pregnancies; and birth weight ≥ 4000 g (macrosomia) (OR 1.7) (Fig. 2). In gestational diabetes the multivariate analysis show significant association with: caesarean section (OR 1.5); moderate (OR 1.7), high (OR 1.7) and very high (OR 1.8) risk pregnancies and lower 1-minuto Apgar score (OR 1.5) (Fig. 3).

## Discussion

In this retrospective study, prevalence of gestational and pregestational diabetes were 6.6% and 1.6%, respectively. Pregestational diabetes in pregnancy is associated with higher rates of prematurity, caesarean section and macrosomia, and gestational diabetes is associated with caesarean section, lower 1-min Apgar scores and risk of pregnancy (moderate, high and very high).

Preterm births account for 75% of neonatal mortality and almost 50% of long-term neurological morbidity^10–13^. In this study, the prevalence of preterm births was 8.6% in patients with diabetes compared to 5.7% in patients without diabetes. Similarly, a case–control study found a higher incidence of foetal distress, macrosomia, small for gestational age and preterm infants in mothers with GD compared to the control group^14^.

Furthermore, a meta-analysis that evaluated the effects of glucose intolerance (GI) that does not reach the criteria for gestational diabetes, observed an increase in caesarean sections, babies large for gestational age (LGA), preeclampsia, preterm births and low Apgar scores in women with GI^15^.

Regarding the increased risk of caesarean section, the Hyperglycaemia and Adverse Pregnancy Outcome (HAPO) study reported an increased risk in women with elevated glucose concentrations^4^. Analysing the characteristics and outcomes of pregnant women with gestational diabetes according to insulin sensitivity, Benhalima et al.^16^ observed that patients with gestational and insulin-resistant diabetes presented higher rates of preterm births (8.5% vs. 4.7%, p = 0.030), need for induction of labour (42.7% vs. 28.1%, p < 0.001), total caesarean sections (28.7% vs. 19.4%, p = 0.00) and emergency caesarean sections (16.0% vs. 9.7%, p = 0.010) compared with women without diabetes. In the current study, the proportion of caesarean sections was also higher in women with than without diabetes (25.4% vs. 16.6%, p < 0.001).

Our data agree with the literature, although the percentage of caesarean sections varies in different studies. Moore et al.^17^ analyse the cultural perception of the caesarean section and suggest that it is possible to reduce its rates. Powe et al.^18^ analyse gestational diabetes in relation to insulin sensitivity, reporting a higher number of caesarean sections (33.3% vs 15.2%) in patients with impaired insulin sensitivity, even after adjusting for BMI.

Regarding Apgar scores < 7, they have been associated with gestational and pregestational diabetes^5,19,20^ in various studies, and with higher rates of respiratory distress and admission to neonatal intensive care units. Preda et al.^18^ reports a 1-min Apgar < 7 in 7.8% of mothers with GD compared to 0% in mothers without GD.

Other studies have also shown macrosomia as a significant adverse effect in gestational and pregestational diabetes^21–24^. The longitudinal Lawlor study associated diabetes with macrosomia^25^. Zeng et al.^26^ corroborated the association between gestational and pregestational diabetes with macrosomia. Since they found a significant association between glycaemia one year before pregnancy and macrosomia, they proposed to perform a rapid glucose test during check-ups in women before they become pregnant. Other factors also associated with macrosomia are obesity, age of the pregnant mother, body mass index, hypertension, and smoking^8,27^.

## Limitations

We believe that the sample obtained from a large population database has avoided a possible selection bias in this observational study. Since this is a retrospective study, some variables might not be well recorded in the medical history, i.e., socio-demographic data, and data regarding control of diabetes during pregnancy which can be related to complications, such as HbA1c or glycaemic control. It is currently unclear if current GD treatment guidelines can completely prevent long-term adverse effects. However, some studies have shown that treatment of GD based on different thresholds can reduce the incidence of macrosomia and other unfavourable perinatal outcomes^28–31^.

In our study, we have not differentiated the different subtypes of gestational diabetes. This is relevant, since we observe that women with high insulin resistance show worse metabolic parameters during complications of pregnancy, while the phenotype and outcomes of women with insulin-sensitive gestational diabetes are more similar to women without diabetes^16^. We believe that these clinical phenotypes may also be associated with the complications of gestational diabetes.

We have analysed the outcomes in relation to diabetes during pregnancy, however undertaking subgroup analyses, considering factors such as BMI, age, and ethnicity, could provide more detailed insights into the risks linked with gestational and pregestational diabetes.

Despite these limitations, since we have extracted a large sample from a universal health system, we believe that our data reliably reflect the complications of diabetes in our environment.

## Conclusions

In conclusion, screening and diagnosis of diabetes mellitus during and before pregnancy is crucial for an appropriate management that prevents maternal and neonatal complications and reduces the chronic cardio-metabolic risk of mother and child.

Our study shows that pregestational and gestational diabetes correlate with a larger incidence of prematurity, caesarean sections, worse 1-min Apgar scores, and macrosomia.

Obesity and diabetes continue a global upward trend with negative effects on patients and society. Adhering to clinical guidelines holds significant importance for clinicians, facilitating early counseling to pregnant women concerning risk factors and requisite interventions in diabetes mellitus^32^.

## Data Availability

The data used in this study are only available for the participating researchers, in accordance with current European and national laws. Thus, the distribution of the data is not allowed. However, researchers from public institutions can request data from SIDIAP. Further information is available online (https://www.sidiap.org/index.php/en/solicituds-en).
